# Exploiting Nanoscale Complexion in LATP Solid-State Electrolyte via Interfacial Mg^2+^ Doping

**DOI:** 10.3390/nano12172912

**Published:** 2022-08-24

**Authors:** Sina Stegmaier, Karsten Reuter, Christoph Scheurer

**Affiliations:** 1 Department of Chemistry, Technical University of Munich, 85747 Garching, Germany; 2 Theory Department, Fritz-Haber-Institut der Max-Planck-Gesellschaft, 14195 Berlin, Germany; 3 Institute of Energy and Climate Research, Fundamental Electrochemistry (IEK-9), Forschungszentrum Jülich GmbH, 52425 Jülich, Germany

**Keywords:** complexion, interface engineering, cationic doping, protective coating, solid state electrolyte, molecular dynamics

## Abstract

While great effort has been focused on bulk material design for high-performance All Solid-State Batteries (ASSBs), solid-solid interfaces, which typically extend over a nanometer regime, have been identified to severely impact cell performance. Major challenges are Li dendrite penetration along the grain boundary network of the Solid-State Electrolyte (SSE) and reductive decomposition at the electrolyte/electrode interface. A naturally forming nanoscale complexion encapsulating ceramic Li${}_{1+x}$Al${}_{x}$Ti${}_{2-x}$(PO${}_{4}$)${}_{3}$ (LATP) SSE grains has been shown to serve as a thin protective layer against such degradation mechanisms. To further exploit this feature, we study the interfacial doping of divalent Mg${}^{2+}$ into LATP grain boundaries. Molecular Dynamics simulations for a realistic atomistic model of the grain boundary reveal Mg${}^{2+}$ to be an eligible dopant candidate as it rarely passes through the complexion and thus does not degrade the bulk electrolyte performance. Tuning the interphase stoichiometry promotes the suppression of reductive degradation mechanisms by lowering the Ti${}^{4+}$ content while simultaneously increasing the local Li${}^{+}$ conductivity. The Mg${}^{2+}$ doping investigated in this work identifies a promising route towards active interfacial engineering at the nanoscale from a computational perspective.

## 1. Introduction

Secondary batteries as intermittent storage devices, have proven to be an indispensable component to advance in the ongoing energy transition from fossil fuels to more sustainable alternatives. State-of-the-art Lithium Ion Batteries (LIBs) in current commercial applications featuring a liquid based electrolyte are expected to gradually be replaced in the upcoming years by a next generation of All Solid-State Batteries (ASSBs) [1,2]. By replacing the highly flammable organic liquid with a Solid-State Electrolyte (SSE), ASSBs promise improved operation safety as well as higher energy density and cycle lifetime [3,4].

Though bulk SSE materials with competitive ionic conductivities have been developed [5,6], the realization of such multi-component functional solid-state devices is considerably hindered by often fatal interfacial processes. Reactive electrochemical contact instabilities between the electrode and the electrolyte as well as metallic dendrite nucleation and growth through the SSE typically induce cell failure [7,8]. Macroscopic bulk properties of individual components alone do not yield a sufficiently detailed picture of the SSE. Instead, recent studies have demonstrated that the structural and chemical constitution of interfaces at a nanometer scale needs to be considered for a more nuanced picture [9,10,11].

In our previous work we have established a realistic grain boundary atomistic model in the ceramic Li${}_{1.3}$Al${}_{0.3}$Ti${}_{1.7}$(PO${}_{4}$)${}_{3}$ (LATP) electrolyte by leveraging experimental Transmission Electron Microscopy (TEM) and Atom Probe Tomography (APT) findings [9]. A protective nanometer thin interlayer encapsulating the crystalline LATP grains was identified, which effectively acts as a protective coating and mitigates reductive degradation and Li nucleation. The interphase exhibits a thermodynamically stable, self-limiting width of ≈1.4 nm [9] and shows distinct structural and compositional features different from adjacent bulk phases. Such 2D nanostructures have recently been termed complexions [12,13,14,15,16]. It is hypothesized that the local separation of Transition Metal (TM) centers from mobile Li${}^{+}$ charge carriers, mediated by the observed nanoscale complexion, suffices as a sizable electronic impedance, thus protecting the crystalline grains. A detailed understanding of an SSE’s transport properties and stability would thus require an extension of the widely accepted crystalline bulk material assessment by taking nano-motifs such as the found complexion explicitly into account.

Other research fields, i.e., semiconductor physics [17] and nano-ionics [18,19], already actively exploit such local phenomena to design high-performance materials. Interfacial engineering of the manifold of solid-solid interfaces present in any ASSB battery setup has recently become the main focus of an entire research field [20,21]. The majority of these efforts is targeted at engineering electrode/electrolyte interfaces, e.g., by introducing buffer layers as coatings [22] or improved contacting enabled by ultra smooth SSE surfaces [23]. In addition to surfaces which are exposed to the electrodes, also buried interfaces within the SSE are of great interest. Especially in order to suppress dendrite growth and residual electron transport through the network of grain boundaries. Xu et al. [24] have recently shown how mixing of second phase additives into Li${}_{6.5}$La${}_{3}$Zr${}_{1.5}$Ta${}_{0.5}$O${}_{12}$ (LLZTO) mother powders leads to a glassy phase distributed along the grain boundaries, thereby effectively suppressing Li dendrite growth in the SSE. The local altering of chemical composition and structural engineering of interfaces thus effectively resembles a nanomaterial synthesis.

As these interfaces are not exposed, they are experimentally difficult to access and analyze post synthesis. The role of computational methods is therefore crucial to not only understand the interfacial processes mechanistically, but also to predict promising engineering routes for improved local performance. Extensive simulations, mostly at first-principles level, have indeed been conducted on either improving SSE bulk material properties, e.g., via interstitial doping [25,26], or studying electrolyte/electrode interfacial compatibility [27,28,29]. The general premise of such calculations though is the mapping of a representative system into smaller idealized cells to arrive at computationally manageable length and time scales. However, to truly study and even more so, engineer these confined nanostructures in a multi-phase setting, adjacent bulk phases need to be represented in the cell, leading to much larger atomic structures for realistic models.

Building on the previously established realistic grain boundary model for LATP, we here investigate a possible interfacial engineering by cationic doping to further exploit the advantageous properties of the found complexion. For true interfacial engineering of confined nanostructures, several requirements need to be met and will be addressed via classical force field based simulations. First, the interfacial dopant must not penetrate significantly into the crystalline bulk of the electrolyte grains to guarantee long-term stability of the engineered interface. Second, the interfacial doping should not compromise the bulk electrolyte performance and, third, the engineering should exploit and enhance desirable features of the (semi-)amorphous interphase. Motivated by APT findings of local accumulation in the grain boundary streak [9], we will focus on the divalent Mg${}^{2+}$ as a potential dopant candidate and scrutinize the effect of seven different doping concentrations on the postulated requirements. For all tested doping concentrations we in fact find the Mg${}^{2+}$ to mostly stay spatially confined in the grain boundary domain, and thus not severely compromising the electrolyte performance. The protective nature of the nano-scale complexion in LATP could be exploited via interfacial doping by reducing residual local electronic conductivity while simultaneously improving grain boundary Li ion conductivity.

## 2. Materials and Methods

### 2.1. Molecular Dynamics Simulations

All Molecular Dynamics (MD) Simulations are performed using the LAMMPS Molecular Dynamic Simulator [30] and the herein extended core-shell force field for Mg${}^{2+}$ doped Li${}_{1.3}$Al${}_{0.3}$Ti${}_{1.7}$(PO${}_{4}$)${}_{3}$ (LATP). A short-range interaction cutoff of 9 Å is chosen and 3D periodic boundary conditions are applied. To treat the long-range Coulombic interactions a Particle-Particle-Particle-Mesh solver [31] is used. A small timestep of 0.2 fs is chosen to capture the high frequency core-shell vibrations of the oxygen anions. For simulations in the canonical NVT ensemble a Nose-Hoover thermostat is used as implemented in the LAMMPS software (Large-scale Atomic/Molecular Massively Parallel Simulator, release 12 December 2018, Sandia National Laboratories, Temple University, Philadelphia, PA, USA) [32]. Similarly for the isothermal-isobaric NPT ensemble a Nose-Hoover barostat is used. Relaxation times are adopted as suggested with $T_{\mathrm{damp}}$ = 100 dt and $p_{\mathrm{damp}}$ = 1000 dt, respectively.

### 2.2. Underlying Li_1+x_Al_x_Ti_2−x_(PO_4_)_3_ Core-Shell Force Field

All MD simulations are performed using an underlying core-shell force field previously parameterized for Li${}_{1+x}$Al${}_{x}$Ti${}_{2-x}$(PO${}_{4}$)${}_{3}$ (LATP) [9]. The classical force field is derived from first-principles Density-Functional Theory (DFT) calculations via parameter fitting from energy matching. To ensure a certain degree of flexibility and transferability, the force field was subsequently extended and reparameterized for structurally similar and chemically related LiTi${}_{2}$(PO${}_{4}$)${}_{3}$, AlPO${}_{4}$, LiTiPO${}_{5}$ and LATP. Long-range Coulombic interactions and short-range van der Waals interactions are analytically formulated with a Buckingham potential [33]. To account for the polarization of the phosphate tetrahedra, the oxygen anions are modelled as core-shell particles. The core particle and the satellite shell pseudoparticle are connected via a harmonic spring to allow for core-shell vibration and parameters are adopted from Kerisit et al. [34]. A more comprehensive description of the LATP core-shell force field is provided in the Supporting Information of our previous work [9].

### 2.3. Force Field Extension by Mg^2+^

The previously introduced core-shell force field [9], specifically parameterized for LATP, is extended by Buckingham parameters for the Mg${}^{2+}$-ion interactions. A Particle Swarm Optimizer [35] is employed for global optimization via energy and force matching against first principles doped reference data. The DFT reference calculations are obtained using the CASTEP [36] plane wave code along with the PBE exchange-correlation functional [37] and ultrasoft pseudopotentials as provided by the GBRV library [38]. Converged settings are adopted from previous work [9] with a plane wave cutoff energy of 750 eV and a Monkhorst-Pack grid density [39] of 0.07 Å${}^{-1}$. A comprehensive workflow of the parameterization scheme is provided in the Supporting Information and the final force field parameters are listed in Table A1. Locally optimized initial parameters of Mg–phosphate interactions in Mg${}_{3}$(PO${}_{4}$)${}_{2}$ are listed in Table S1 and partial radial distribution functions shown in Figure S1. Energy and force correlation of the final extended force field and reference DFT data are shown in Figure S2 and ion dynamics retrieved from MD simulations are shown in Figure S3.

### 2.4. Monte-Carlo Swapping Protocol

Following a Monte-Carlo (MC) based protocol recently introduced by Türk et al. [40], Mg${}^{2+}$ is swapped across an interface for Ti${}^{4+}$, Al${}^{3+}$ or Li${}^{+}$. Swapping attempts are accepted according to a Metropolis algorithm with

(1) $$\Delta E:=E_{\mathrm{after}}-E_{\mathrm{before}},$$

if it leads to a gain in potential energy, i.e., ΔE≤0. For ΔE>0, a random number *q* is drawn and the swap is accepted if p>q with

(2) $$p=\exp\left[-\frac{\Delta E}{k_{B}T}\right],$$

where $k_{B}$ is the Boltzmann constant and *T* is the system temperature. The swapping is performed in a layer-wise manner to mimic Fickian diffusion [40] with a layer width of 3.5 Å, which corresponds to the *z*-distance between Ti/Al planes in the crystalline domain. After 100 attempted swaps, short NPT simulations are performed at 1000 K and 1 bar for 2 ps to allow for structural relaxation into local basins of the presumably shallow Potential Energy Surface and for the redistribution of Li${}^{+}$ to maintain local charge neutrality. A new layer is added every 10 such repetitions. With a total of 15 layers, 15,000 attempted swaps are performed and a total relaxation time of 300 ps is simulated. A region of ≈52.5 Å from the interface into the grain is explored. For robust statistical ensembles, multiple such MC walkers are run in parallel and different initial configuration temperatures are exchanged through parallel tempering, a replica exchange method [41,42]. Walker simulations are initialized at equidistant temperatures in a regime between 1000–2000 K. Elevated temperatures are chosen to enhance dynamics. All walkers explore the full temperature regime. Exemplary walker energy convergence and configurational exchange via parallel tempering is schematically depicted in the Supporting Information Figure S4.

### 2.5. Ion Dynamics Analysis

Atomistic motion is translated into macroscopic diffusion applying the Einstein formulation of tracer diffusion via the Mean Square Displacement (MSD) of the ions with

(3) $$\mathrm{MSD}\left(\tau \right)=\langle |\vec{r_{i}}\left(t-\tau \right)-\vec{r_{i}}\left(t\right){|}^{2}\rangle ,$$

where τ is a so-called lag time used for enhanced statistical sampling, $r_{i}$ is the atom position and the angular brackets denote averaging over the number of atoms to get an ensemble property. The tracer diffusion coefficient is then obtained via

(4) $$D^{*}=\frac{1}{3}\frac{\langle |\vec{r_{i}}\left(t-\tau \right)-\vec{r_{i}}\left(t\right){{|}^{2}\rangle }_{\tau }}{2\Delta t}\;,$$

where Δt is the sampling time. This diffusion coefficient can subsequently be translated into an ionic conductivity by means of the Nernst-Einstein relation [43] as

(5) $$\sigma =\frac{1}{6Vk_{B}T}\sum_{\beta }^{n_{\beta }}q_{\beta }^{2}D_{\beta }^{*}N_{\beta },$$

with *V* the cell volume, $k_{B}$ the Boltzmann constant, *T* the system temperature and $q_{\beta }$, $D_{\beta }^{*}$, $N_{\beta }$ the charge, tracer diffusion coefficient and number of particles of species β, respectively. This formulation only holds for linear behavior of the MSD. Following recent findings of He et al. [44], the calculation of diffusivity fitting to the Einstein relation is limited to run times above 10% to exclude the ballistic motion regime and below 70% due to poor linearity from statistics.

## 3. Results

### 3.1. Mg^2+^ as Interfacial Dopant Candidate

**Figure 1:**
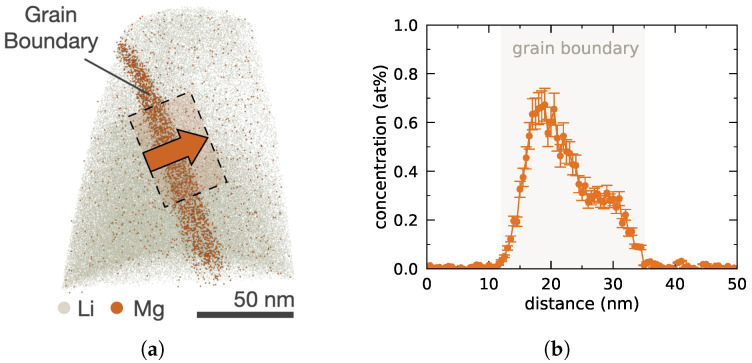
Accumulation of Mg${}^{2+}$ in LATP grain boundary. (**a**) Reconstructed Mg atom map 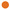 visualizing Mg${}^{2+}$ accumulation in the grain boundary of the APT sample and Li atom map 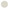 as reference. (**b**) Elemental Mg profile across the grain boundary, where the overlayed rectangle 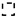 in (**a**) indicates the selected subvolume used for averaging, with the arrow indicating the direction used for positive distances. Adapted with permission from Ref. [9] under CC BY 4.0.

Atom maps retrieved from previous APT analysis of a grain boundary in LATP [9] reveal a local accumulation of divalent Mg${}^{2+}$ in the amorphous grain boundary, cf. Figure 1a, hence suggesting it to be a promising interfacial dopant candidate. With a peak concentration well below 1 at%, cf. Figure 1b, the observed Mg${}^{2+}$ accumulation qualifies as an unintentional impurity. Preferential doping of Mg${}^{2+}$ into interphases rather than bulk grains has recently also been reported by Cheung et al. for the Na${}_{1+x}$Zr${}_{2}$Si${}_{x}$P${}_{3-x}$O${}_{12}$ sodium (NA) Super Ionic CONductor (NASICON) [45].

From an electrochemical perspective, Mg is generally a suitable dopant candidate in LIBs due to its redox stability against metallic Li [46,47]. Additionally, in the specific case of LATP no severe structural destabilization or steric hindrance is expected from Mg${}^{2+}$ doping due to its very similar ionic radius as compared to the SSE-constituting elements [48].

With a formal charge of $q_{\mathrm{Mg}}$ = +2, which is different from all other cations in LATP, Mg${}^{2+}$ allows for aliovalent substitution. From an engineering perspective, this is often exploited in highly ordered materials in order to introduce defined cationic defects which may enhance ion mobility [49]. In amorphous phases, aliovalent doping opens a design route to deliberately alter the cationic composition by e.g., decreasing the fraction of higher valent cations while increasing lower valent ones.

### 3.2. Atomistic Structures of Mg^2+^ Interfacially Doped LATP

**Figure 2:**
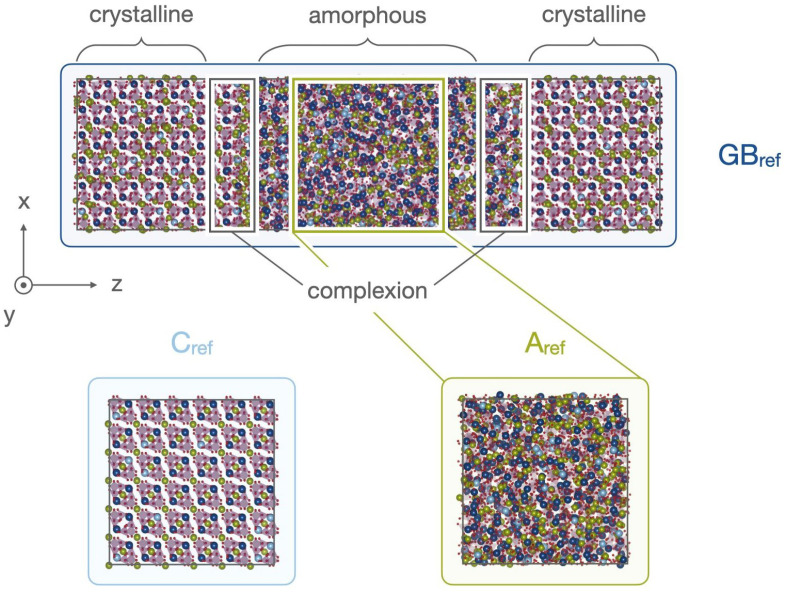
Atomistic structures of LATP grain boundary GB${}_{\mathrm{ref}}$ and representative bulk structures for LATP crystalline slab C${}_{\mathrm{ref}}$ and amorphous bulk slab A${}_{\mathrm{ref}}$. The grain boundary structure is differentiated into three domains: crystalline grains, amorphous bulk and nanoscale complexions encapsulating the grains. The amorphous bulk cells are cut from the respective bulk domain in the respective grain boundary structure. Elemental colors are chosen as Li 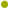, Al 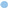, Ti 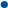, O 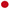, and P 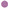. The atomistic grain boundary reference structure is adapted with permission from Ref. [9] under CC BY 4.0.

In order to investigate whether and to what extent Mg${}^{2+}$ interfacial doping is beneficial to the LATP SSE performance, we construct an atomistic reference structure following the computational protocol established in our earlier work [9]. In brief, this protocol yields a realistic LATP structure featuring an extended amorphous grain boundary encompassed by crystalline grains as observed in corresponding TEM studies [9]. While the atomistic built up of crystalline LATP bulk is well known [50,51,52,53], the chemically differing atomistic composition in the amorphous domain is modelled after elemental profiles retrieved from APT analysis. Cations and phosphate units are initially stochastically sampled onto a sparse grid between the grains and an established computational sintering protocol is applied to obtain the final structure. The sintering protocol mimics the experimental procedure via annealing and iterative compression, a short melting sequence and quenching of the structure. A resulting reference grain boundary structure with crystalline, amorphous and identified nanoscale complexion domains is shown in Figure 2 as GB${}_{\mathrm{ref}}$. The reference grain boundary structure [54] comprises a total of 14,030 atoms in a 3D periodically extended cell of 36.24 Å × 36.24 Å × 139.28 Å size. Respective bulk cells of crystalline LATP and amorphous grain boundary bulk are shown as C${}_{\mathrm{ref}}$ and A${}_{\mathrm{ref}}$.

The three structurally different domains of crystalline LATP grain, nanoscale complexion, and amorphous grain boundary can be clearly differentiated from 2D Fourier analysis of the distortion within the Ti-Al submanifold. Inspired by the APT findings, Mg${}^{2+}$ is initially doped into the amorphous bulk domain of the reference GB${}_{\mathrm{ref}}$ only. Maintaining system charge neutrality, Mg${}^{2+}$ is substituted for either

(6) $$\begin{array}{cc} 1\;{\mathrm{Ti}}^{4+} & ⟶1\;{\mathrm{Mg}}^{2+}+\;2\;{\mathrm{Li}}^{+},\mathrm{or}\\ 1\;{\mathrm{Al}}^{3+} & ⟶1\;{\mathrm{Mg}}^{2+}+\;1\;{\mathrm{Li}}^{+}. \end{array}$$

Though other charge neutral substitutions are possible, these are chosen first, to deliberately reduce the Ti${}^{4+}$ content and second, to simultaneously increase the Li${}^{+}$ content. A decrease of Ti${}^{4+}$ TM centers is desirable to reduce residual electronic conductivity via polaron hopping [55,56]. The simultaneous increase in Li concentration may favorably impact the grain boundary ion conductivity as recently suggested by Mertens et al. [57]. While the first substitution in Equation (6) suffices to reduce TMs, the introduction of three new particles leads to a substantially higher particle count in the amorphous grain boundary. This could potentially lead to drastic effects on structural integrity of the host system. To mitigate this risk, both Al${}^{3+}$ and Ti${}^{4+}$ are substituted. An introduction of charged defect pairs is explicitly not realized as the highly mobile Li${}^{+}$ will compensate for local charge effects. The interaction of such defects has been shown to be extremely short-range and significantly shorter than pure electrostatic screening [58].

**Figure 3:**
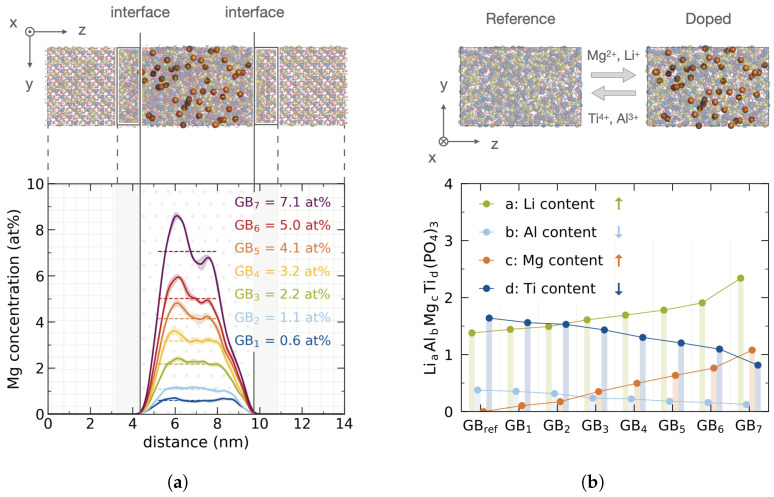
Initial Mg${}^{2+}$ doped structures of the LATP grain boundary reference. (**a**) Top: Atomistic structure with elemental colors chosen as Li 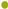, Mg 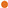, Al 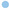, Ti 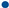, O 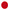, and P 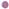; (for better visualization Mg${}^{2+}$ is depicted larger and other ions are transparent). Bottom: Corresponding Mg${}^{2+}$ profiles across the grain boundary for different doping concentrations GB${}_{1}$–GB${}_{7}$. (**b**) Top: Amorphous bulk reference structure and exemplary doped structure. Elemental colors, radii and opacity are adopted from (**a**). Bottom: Cationic composition normalized to phosphate content with Li${}_{a}$Al${}_{b}$Mg${}_{c}$Ti${}_{d}$(PO${}_{4}$)${}_{3}$.

The compositions studied herein range from amorphous bulk concentrations of 0.6 at%–7.1 at% for GB${}_{1}$–GB${}_{7}$, respectively. The underlying reference structure GB${}_{\mathrm{ref}}$ is the same for all doping realizations. The lower doping limit in GB${}_{1}$ is chosen to reproduce the Mg${}^{2+}$ content found in the experimental LATP grain boundary. The upper limit in GB${}_{7}$ is chosen to reduce the Ti${}^{4+}$ content to half the reference. An exemplary atomistic structure highlighting the dopant in the amorphous domain and respective Mg${}^{2+}$ profiles across the grain boundary are shown in Figure 3a. Since the doping realizations are stochastic in nature, each composition GB${}_{1}$–GB${}_{7}$ is sampled 12 times and relaxed after application of a MC swapping protocol, cf. Section 2.4. The six energetically most favorable configurations are taken as a composition ensemble.

Doping is performed following the experimentally reported APT profiles resulting in two Mg${}^{2+}$ concentration maxima as shown in Figure 3a. By design the Mg${}^{2+}$ concentration in the crystalline grain domain is set to zero, yielding an initial structure with true interfacial doping. Corresponding cationic compositions in the amorphous bulk for each of the seven doping concentrations are shown in Figure 3b. The fraction for each cation is normalized by the amount of phosphate units according to Li${}_{a}$Al${}_{b}$Mg${}_{c}$Ti${}_{d}$(PO${}_{4}$)${}_{3}$. Normalization is consistent across all configurations as a common reference structure GB${}_{\mathrm{ref}}$ is chosen. Following the cationic substitutions outlined in Equation (6), both Mg${}^{2+}$ and Li${}^{+}$ contents are raised, while Ti${}^{4+}$ and Al${}^{3+}$ contents are decreasing to half the reference content, i.e., for Al${}^{3+}$ from b${}_{{\mathrm{GB}}_{\mathrm{ref}}}$ = 0.4 to b${}_{{\mathrm{GB}}_{7}}$ = 0.2 and for Ti${}^{4+}$ from d${}_{{\mathrm{GB}}_{\mathrm{ref}}}$ = 1.6 to d${}_{{\mathrm{GB}}_{7}}$ = 0.8.

MD Simulations in NPT are performed for 100 ps at 300 K and 1 bar for each realization to allow for energetic relaxation of the initially doped structures. These simulations employ a previously introduced classical core-shell force field for Li${}_{1+x}$Al${}_{x}$Ti${}_{2-x}$(PO${}_{4}$)${}_{3}$ [9], which has been extended by Mg${}^{2+}$ interaction terms, c.f. Section 2.2 and Section 2.3.

### 3.3. Dopant Bleeding into Grain Bulk Domain

While a strict spatial separation is desired for a true interfacial dopant, aging processes as well as ion diffusion may lead to bleeding, i.e., leakage, of Mg${}^{2+}$ into the crystalline grain domains, thus escaping the intended interfacial engineering purpose.

A direct simulation of such processes, which can take days, months and years of cycling in real time [59,60,61], exceeds the computationally accessible time scales by multiple orders of magnitude. We therefore adopt and adjust a recently introduced statistical sampling scheme by Türk et al. [40] to assess possible (inter-)diffusion processes across interfaces in our simulations. To briefly outline the sampling protocol, Mg${}^{2+}$ ions are randomly swapped for Ti${}^{4+}$, Al${}^{3+}$ or Li${}^{+}$ cations across the interface. A swap is accepted if energetically favorable according to a Metropolis MC criterion based on potential energy. Recurring after 100 swapping attempts, short MD simulations are employed to allow the structure to relax into local basins of the shallow potential energy surface and to allow local charge compensation for introduced lower valent Mg${}^{2+}$. The dopant may thus penetrate into the crystalline grain in a layer-wise fashion, mimicking Fickian diffusion [40]. Since this is a stochastic process, multiple of such randomly initialized swapping walkers are simulated in parallel, exchanging configurations at different temperatures as given by the replica exchange method [41,42]. As a result, a robust ensemble of configurations exploring low and high energy configurations is obtained of which only the lowest six energy walkers are considered for further investigations. The MC swapping protocol is elaborated in more detail in Section 2.4.

**Figure 4:**
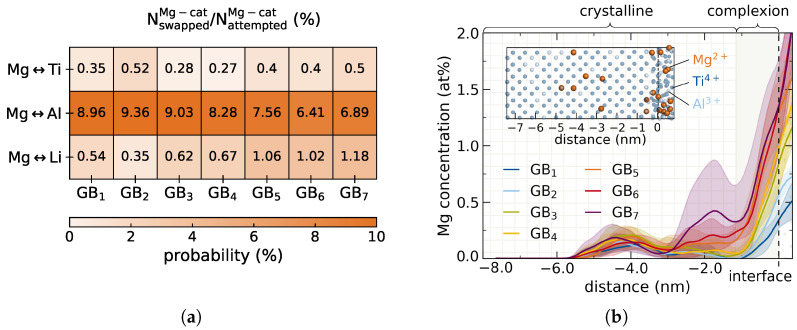
Penetration of Mg${}^{2+}$ into LATP grain after MC swapping. (**a**) Normalized acceptance probabilities of swappings for each Mg ↔ cation pair in all doping concentrations GB${}_{1}$–GB${}_{7}$ with N${}_{\mathrm{swapped}}^{\mathrm{Mg}–\mathrm{cat}}$/N${}_{\mathrm{attempted}}^{\mathrm{Mg}–\mathrm{cat}}$. (**b**) Mg${}^{2+}$ profiles across grain boundary after MC swapping averaged over the six lowest energy walkers for each doping concentrations. An exemplary atomistic substructure of crystalline Ti–Al framework with incorporated Mg${}^{2+}$ ions after swapping is shown.

Figure 4a depicts the acceptance probabilities of a Mg–cation swap for each cationic species averaged over all walkers for each doping composition GB${}_{1}$–GB${}_{7}$. The analysis shows a clear preference of Mg${}^{2+}$ swapping for Al${}^{3+}$, as compared to Ti${}^{4+}$ or Li${}^{+}$, with an order of magnitude higher success rates for accepted Mg ↔ Al swaps. While the Mg–Al acceptance probabilities of ≈6.41–9.36% seem large in comparison to the other cations, the absolute number of Mg incorporated onto Al sites in the crystal domain remains low due to the low absolute Al content in Li${}_{1+x}$Al${}_{x}$Ti${}_{2-x}$(PO${}_{4}$)${}_{3}$ with x = 0.3. Due to a normalization effect, the Mg ↔ Al acceptance probabilities exhibit a decrease with increasing doping concentration, since the Al${}^{3+}$ content is deliberately reduced. Only when increasing the doping concentration, swapping of Mg${}^{2+}$ onto Ti${}^{4+}$ and Li${}^{+}$ sites becomes more probable. This can be interpreted as a result of the thermodynamic driving force due to a higher chemical potential when more Mg${}^{2+}$ ions are spatially confined in the amorphous region.

Translating the obtained Mg–cation swapping acceptance probabilities into quantifiable dopant bleeding, Figure 4b shows the resulting Mg${}^{2+}$ concentration profiles across the interface after MC swapping. The atomistic models feature two interfaces between the crystalline grain and the amorphous grain boundary due to periodic boundary conditions used in the simulations. Yet, MC swapping is performed asymmetrically across only one interface. Limiting the bleeding analysis to one interface is crucial since the crystalline bulk domain in the simulated system does not extend far enough to avoid overlapping of the Mg leakage from both sides. The system size is limited to maintain a manageable computational cost. In real systems the leakage of dopant is expected to occur symmetrically at both interfaces.

As can be seen from Figure 4b, Mg${}^{2+}$ ions penetrate about ≈4 nm into the crystal beyond the complexion. This nm ranged penetration into the crystal lattice is considered a localized phenomenon compared to the μm range cross section of typical physical LATP grains reported from experiment [57,62]. The Mg-profiles in Figure 4b suggest an initial higher amorphous bulk Mg${}^{2+}$ content leading to increased bleeding into the crystal. However, for all doping compositions, the amount of Mg${}^{2+}$ incorporated into the crystal is significantly lower than the amorphous bulk concentration. For the lower doping concentrations this translates into single Mg${}^{2+}$ ions being swapped into the crystal lattice. A high success rates above 9% for Mg ↔ Al in GB${}_{1}$ does not necessarily result in higher Mg${}^{2+}$ concentration in the grain, as the MC protocol accounts for re-swapping into the amorphous bulk. Mg${}^{2+}$ concentrations below 0.5 at% further into the crystal are within the impurity range as observed experimentally in previous work [9].

**Figure 5:**
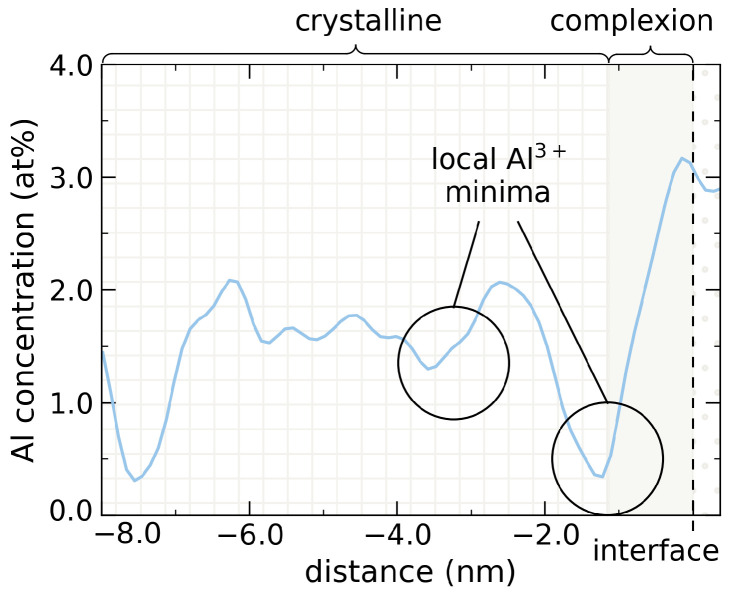
Al concentration profile of the undoped reference system with local concentration minima at −1.1 nm and −3.2 nm from the interface. As Mg${}^{2+}$ preferentially occupies former Al${}^{3+}$ sites, these local minima are also observed for the dopant bleeding in Figure 4b.

All Mg-profiles in Figure 4b after MC swapping exhibit two more or less pronounced concentration minima at −3.1 nm and −1.1 nm from the swapping interface. These minima correspond to local concentration drops in the Al-profile of the underlying host reference system, cf. Figure 5.

A characteristic depletion of Al${}^{3+}$ resulting from a reported Ti–Al segregation in the complexion [9] therefore protects the electrolyte grains from Mg${}^{2+}$ penetration. The resulting minimal bleeding even at high doping concentrations thus meets the first postulated requirement for successful interfacial engineering.

### 3.4. Bleeding Implications on Crystalline Bulk

**Figure 6:**
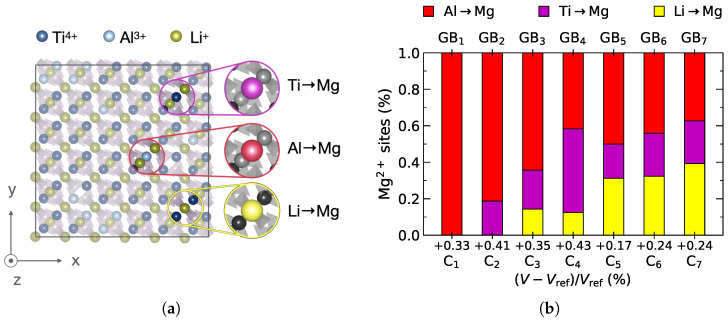
Incorporated Mg${}^{2+}$ in crystalline grain from dopant bleeding simulation. (**a**) Atomistic structure of reference crystal cell C${}_{\mathrm{ref}}$ with possible Mg${}^{2+}$ swapping sites. (**b**) Composition of Mg${}^{2+}$ site occupancy in crystal lattice after MC swapping for different doping concentrations GB${}_{1}$–GB${}_{7}$. Swapping colors are chosen as Mg${}^{2+}\to$ Al${}^{3+}$ 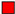, Mg${}^{2+}\to$ Ti${}^{4+}$ 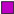, and Mg${}^{2+}\to$ Li${}^{+}$ 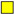. On the abscissa, the relative volume change ($V_{i}-V_{\mathrm{ref}}$)/$V_{\mathrm{ref}}$ in percent, for the correspondingly doped crystal structures C${}_{1}$–C${}_{7}$ is shown.

Quantitatively, the amount of Mg${}^{2+}$ incorporated into the crystalline LATP lattice is low. Nevertheless, even a very thin surfacial region with high ionic impedance might dramatically affect the overall performance. Two conceptually different aspects need to be considered when incorporating an ion into the crystalline LATP. First the Mg${}^{2+}$ substitution into the immobile host structure, i.e., the Ti/Al framework, and second the penetration into the charge carrier Li pathways. Possible Mg${}^{2+}$ swapping sites are shown in Figure 6a.

A site analysis of the incorporated Mg${}^{2+}$ ions into the crystal lattice of the grain boundary structures GB${}_{1}$–GB${}_{7}$, shown in Figure 6b, reveals that the dopant predominantly occupies former Al${}^{3+}$ and Ti${}^{4+}$ sites. Only with rising doping concentration in the amorphous bulk, as in models GB${}_{3}$–GB${}_{7}$, the Li channels are starting to get infiltrated with 11–40% of the Mg${}^{2+}$ ions bleeding into the crystal. In line with the normalized elemental swapping acceptance probabilities, Mg ions exhibit preferential occupation of Al${}^{3+}$ sites specifically for low doping concentrations. Due to the much higher absolute concentrations of Ti${}^{4+}$ and Li${}^{+}$ in LATP though, even low acceptance swapping probabilities lead to incorporation of some Mg${}^{2+}$ also into these sites.

Bleeding of Mg${}^{2+}$ dopant into the Ti/Al host structure may have detrimental implications on the crystalline LATP performance, if it causes structural destabilization of the electrolyte. Since the effective ionic radius of Mg${}^{2+}$ with 72.0 pm [48] is very similar to Ti${}^{4+}$ with 74.5 pm [48], a fatal steric destabilization is highly unlikely. This is further corroborated considering the very low amount of actual Mg${}^{2+}$ bleeding.

To substantiate this hypothesis, the volume change of doped crystalline LATP bulk cells as compared to a reference C${}_{\mathrm{ref}}$ cell is monitored. The underlying C${}_{\mathrm{ref}}$ is cut from reference LATP containing 3953 atoms. The number of Mg${}^{2+}$ atoms for doping is retrieved from summing over the concentration profile in the crystalline domain of the lowest six energy walkers, see Figure 4b, with $\sum x_{\mathrm{Mg}}\cdot N_{C_{\mathrm{ref}}}^{\mathrm{total}}$ = $N_{\mathrm{Mg}}$. Distribution of these atoms onto respective lattice sites is sampled following the respective composition in Figure 6b. Doped crystalline structures for each concentration C${}_{1}$–C${}_{7}$ are obtained after equilibration at 300 K and 1 bar for 100 ps in the NPT ensemble.

A maximum volume change of merely +0.43% is observed for C${}_{4}$ as compared to the pristine LATP reference cell, cf. Figure 6b. The slight volumetric change does not support severe structural changes of the LATP crystal when incorporating Mg${}^{2+}$. Other computational [26] and experimental [63] works further support that Mg${}^{2+}$ doping into the framework of NASICON-type electrolytes does not have a destabilizing impact on the host structure but may even enhance electrolyte performance. Recent studies suggest that even larger ions such as In${}^{3+}$, with an ionic radius of 80.0 pm [48], doped onto Ti${}^{4+}$ sites may in fact stabilize the LATP electrolyte [64].

Bulk LATP is a known ionic superconductor for Li${}^{+}$ [57] due to inherent interconnected 3D diffusion pathways [53,65]. Mg${}^{2+}$ penetrating into these channels may lead to clogging of the charge carrier migration routes.

**Figure 7:**
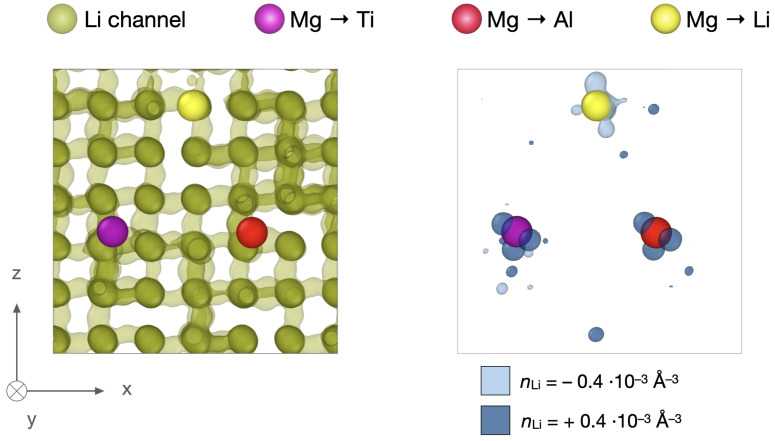
Qualitative assessment of implications of Mg${}^{2+}$ incorporation on Li${}^{+}$ pathways. Left: C${}_{4}$ atomistic structure showing doped Mg${}^{2+}$ ions on former Li${}^{+}$ 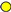, Al${}^{3+}$ 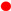, and Ti${}^{4+}$ 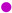 site. Charge carrier 3D pathways are illustrated from collapsing Li${}^{+}$ ions of a 2 ns MD trajectory at 700 K into the same structure 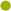. Right: Li density difference of doped system and undoped reference structure showing Li${}^{+}$ avoiding the channel with incorporated Mg${}^{2+}$ at a number density of 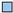 $\Delta n_{\mathrm{Li}}$ = −0.4 10${}^{-3}$Å${}^{-3}$ and Li${}^{+}$ being trapped around Mg${}^{2+}$ on Ti/Al sites with a number density of 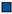 $\Delta n_{\mathrm{Li}}$ = +0.4 10${}^{-3}$Å${}^{-3}$.

For a qualitative assessment of possible clogging, extensive 2 ns NVT simulations at elevated temperatures of 700 K of the doped bulk crystalline LATP structures C${}_{1}$–C${}_{7}$ are conducted. Figure 7 shows an exemplary atomistic structure of C${}_{4}$ with three doping positions. One is a former Li${}^{+}$ site (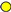 Mg${}^{2+}$ → Li${}^{+}$), another one a former Al${}^{3+}$ site (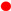 Mg${}^{2+}$ → Al${}^{3+}$) and the third a former Ti${}^{4+}$ site (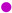 Mg${}^{2+}$ → Ti${}^{4+}$). Li positions of the full 2 ns trajectory are projected onto the same atomistic structure and the isosurface of the resulting Li${}^{+}$ density reveals the interconnected pathways.

Subtraction of the reference Li${}^{+}$ density in the respective LATP bulk C${}_{\mathrm{ref}}$ from the doped structure, yields a change in Li${}^{+}$ density around the doped Mg${}^{2+}$ as exemplarily shown for C${}_{4}$ in Figure 7. As expected, a negative change in Li${}^{+}$ density around the Mg ion in the charge carrier pathway suggests local clogging of the channel. A positive change in Li${}^{+}$ density is observed both, around the Mg ion on Al${}^{3+}$ and Ti${}^{4+}$. This ion “trapping” of charge carriers around cationic constituents *X* doped onto the Ti framework of L*X*TP has been observed in previous experimental [66] and first-principle [67] studies and has been attributed to minor distortions of the LiO${}_{6}$ octahedra due to a difference in ionic radius of the dopant *X*. Additionally, in the case of aliovalent doping here, such doping situations formally constitute negatively charged defects of the host lattice that will be compensated by the density of mobile Li${}^{+}$ on average, i.e., $\Delta q_{{\mathrm{Mg}}^{2+}↔{\mathrm{Ti}}^{4+}}$ = −2 and $\Delta q_{{\mathrm{Mg}}^{2+}↔{\mathrm{Al}}^{3+}}$ = −1. NPT simulations in the MC swapping protocol ensure Li${}^{+}$ redistribution around these defects.

Qualitatively, the bleeding of Mg${}^{2+}$ into charge carrier diffusion pathways does lead to clogging. Additionally the LATP performance may further be compromised by ion trapping around Mg ions doped onto the Ti/Al-host framework. Yet, according to our Li${}^{+}$ density analysis, both effects seem to be extremely localized within the 3D ion channel network.

**Figure 8:**
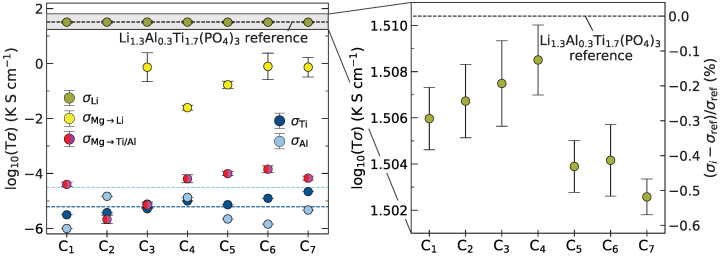
Quantitative assessment of implications of Mg${}^{2+}$ bleeding on ion diffusion. Li${}^{+}$, Mg${}^{2+}$, Al${}^{3+}$, and Ti${}^{4+}$ conductivities as determined from MD simulations at 700 K for C${}_{1}$–C${}_{7}$. Mg${}^{2+}$ conductivities are separated into different site occupation. No Mg${}^{2+}$ is incorporated onto Li${}^{+}$ sites for the lowest two doping concentrations C${}_{1}$ and C${}_{2}$, hence there is no data for these configurations. Li${}^{+}$ conductivities are compared to undoped bulk LATP reference C${}_{\mathrm{ref}}$.

To quantify how such local trapping of charge carriers and clogging of the respective pathways affect the overall performance of the electrolyte, cationic Li${}^{+}$, Mg${}^{2+}$, Al${}^{3+}$ and Ti${}^{4+}$ conductivities from the 2 ns trajectories of C${}_{1}$–C${}_{7}$ are shown in Figure 8. The macroscopic conductivities are obtained by converting atomistic MSDs following the Nernst-Einstein relation as described in Section 2.5. Figure 8 displays the Mg${}^{2+}$ mobility at 700 K to be 6–7 orders of magnitude lower than Li${}^{+}$ for Mg${}^{2+}$ doped onto the Ti/Al framework (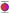). This is similar to the Ti${}^{4+}$ (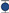) and Al${}^{3+}$ (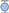) conductivity on the same lattice sites. For Mg${}^{2+}$ doped into Li channels (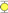), the conductivity is 2–3 orders of magnitude lower as compared to Li${}^{+}$ in C${}_{\mathrm{ref}}$. Even at elevated temperatures the Mg ions are virtually immobile and are thus not expected to penetrate further into the grain with electrolyte aging.

The Li${}^{+}$ conductivity (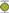) in the doped crystal structures does not seem to be significantly affected. Bleeding of Mg${}^{2+}$ does lead to a decrease in Li${}^{+}$ conductivity, more so for high doping concentrations C${}_{5}$–C${}_{7}$ where Li channels are more heavily infiltrated. Yet, even the largest decrease is merely −0.51% of the observed reference. Therefore, due to the very little bleeding of the dopant, the crystalline bulk performance of LATP grains is not appreciably compromised, encouraging interfacial doping of LATP with the divalent Mg${}^{2+}$.

### 3.5. Interphase Modifications via Aliovalent Doping

The suitability of divalent Mg${}^{2+}$ as a true interfacial dopant has been established from local confinement in the interphase. We now proceed to show that Mg${}^{2+}$ doping can be furthermore exploited to promote desirable features of the recently discovered, particular LATP grain boundary nano-motifs [9].

**Figure 9:**
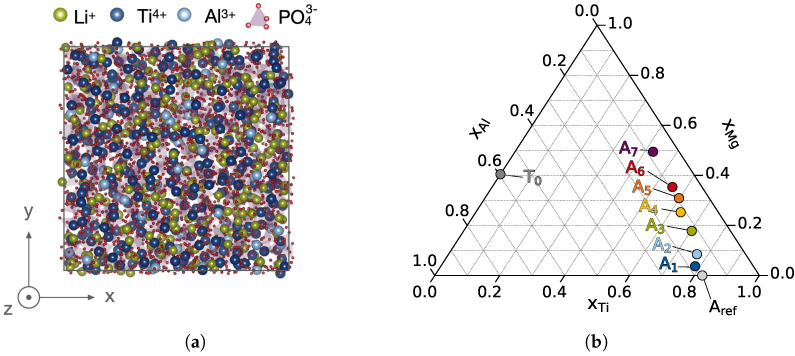
Reference amorphous structure and relative composition of sampled doping stoichiometries. (**a**) Atomistic structure of the reference cell A${}_{\mathrm{ref}}$. Elemental colors are chosen as Li 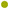, Al 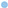, Ti 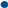, O 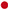, and P 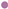. (**b**) Ternary plot showing relative composition for A${}_{\mathrm{ref}}$, A${}_{1}$–A${}_{7}$, and a completely Ti${}^{4+}$ depleted T${}_{0}$ according to $x_{\mathrm{el}}=N_{\mathrm{el}}/\sum \left[N_{\mathrm{Ti}},N_{\mathrm{Al}},N_{\mathrm{Mg}}\right]$.

In order to investigate the effect of Mg${}^{2+}$ doping on properties of the amorphous bulk at manageable computational cost, a smaller amorphous cell is cut from the multi-phase structure GB${}_{\mathrm{ref}}$ to yield a charge neutral and 3D periodic structure A${}_{\mathrm{ref}}$ as shown in Figure 9a. Respective cutting of the amorphous domain of the six lowest energy walkers from GB${}_{1}$–GB${}_{7}$ yields the amorphous cells A${}_{1}$–A${}_{7}$ comprised of 3430–3900 atoms.

The sampled design space is visualized in Figure 9b, where the cation fractions after MC swapping of the Mg-doped LATP (LAMTP) system are normalized with respect to the heavier cations Ti${}^{4+}$, Al${}^{3+}$, and Mg${}^{2+}$ with $x_{\mathrm{el}}=N_{\mathrm{el}}/\sum \left[N_{\mathrm{Ti}},N_{\mathrm{Al}},N_{\mathrm{Mg}}\right]$. The respective Li${}^{+}$ content is dictated by these cations to maintain charge neutrality, as a common underlying reference with a constant total number of phosphate anions is used. By increasing the dopant concentration $x_{\mathrm{Mg}}$, the Al${}^{3+}$ and more importantly the Ti${}^{4+}$ content is reduced to approximately half the amorphous reference, i.e., from $x_{\mathrm{Ti}}^{A_{\mathrm{ref}}}$ = 0.82 to $x_{\mathrm{Ti}}^{A_{7}}$ = 0.42.

Conceptually, in order to minimize electronic conductivity, a complete Ti${}^{4+}$ removal from the interphase seems desirable. To test this extreme case, the reference amorphous bulk is doped with Mg${}^{2+}$ to exchange all Ti ions of the system resulting in configuration T${}_{0}$. Due to a generally higher Mg${}^{2+}$ mobility as compared to Ti${}^{4+}$ and Al${}^{3+}$, the amount of redox stable Al${}^{3+}$ is also increased to maintain the solid nature of the amorphous phase. All amorphous bulk structures are equilibrated in NPT and production runs for ion dynamics studies are conducted in NVT at 700 K for 2 ns.

#### 3.5.1. Dopant Impact on Structural Features

Among other major mechanisms, e.g., penetration through macroscopic defects such as voids or pores [68], dendrite growth in SSEs has been attributed to high local electronic conductivity, directly reducing Li${}^{+}$ ions to metallic Li [7,69,70]. Suppression of Li(0) nucleation becomes even more crucial in the grain boundary region as these form networks that provide an often easily accessible route for further dendrite growth and penetration [7,71]. Consequently, a local minimization of electronic conductivity in the amorphous interphase is highly desirable to further suppress slow degradation processes. Targeted lowering of the content of the reducible Ti${}^{4+}$ via aliovalent doping with Mg${}^{2+}$ may be exploited to increase electronic impedance by influencing polaron hopping pathways.

**Figure 10:**
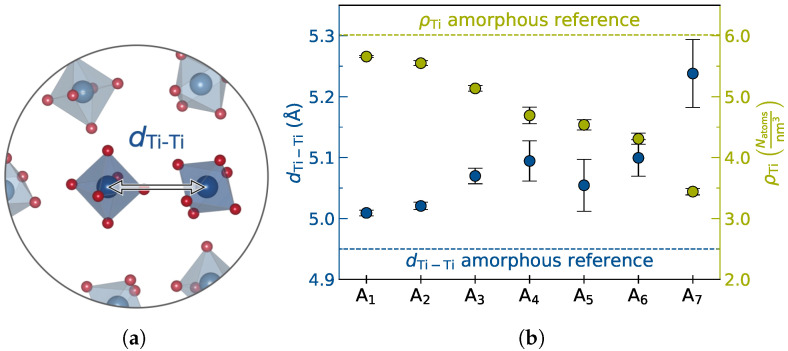
Effect of Mg${}^{2+}$ doping on Ti${}^{4+}$ distribution. (**a**) Zoomed-in atomistic structure depicting Ti-Ti nearest-neighbor (NN) distance $d_{\mathrm{Ti}-\mathrm{Ti}}$. Elemental colors are chosen as Ti 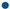 and O 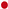. (**b**) $d_{\mathrm{Ti}-\mathrm{Ti}}$ as a function of doping concentration A${}_{1}$–A${}_{7}$ and respective Ti atom density $\rho_{\mathrm{Ti}}$ averaged over six lowest energy walkers defined as $N_{\mathrm{Ti}}/V_{\mathrm{cell}}$.

Figure 10b reveals the average Ti-Ti NN distance $d_{\mathrm{Ti}-\mathrm{Ti}}$ to increase with higher doping concentration. In the highest doping concentration A${}_{7}$ a Ti-Ti NN distance of 5.24 Å is observed which is about 0.3 Å larger than the reference amorphous bulk distance determined as 4.95 Å [9]. In good agreement with the Ti-Ti NN distance of 5.41 Å observed in the protective complexion from previous work [9], substitution of Mg${}^{2+}$ for Ti${}^{4+}$ centers thus is expected to impose an anisotropic electronic impedance disrupting electronic conduction via polaron hopping.

#### 3.5.2. Non-Trivial Effects with Doping Concentration

The trend of increasing the Ti-Ti NN distance is not strictly monotonous or linear with doping concentration. For A${}_{5}$ a slight dip is observed. This non-trivial effect is reflected to a lesser extent in the Ti atom density $\rho_{\mathrm{Ti}}$ of the doped system shown in Figure 10b.

**Figure 11:**
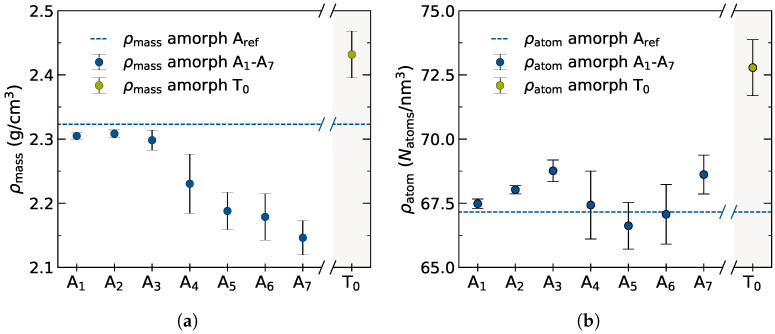
Non-linear effects when doping the amorphous phase with Mg${}^{2+}$. (**a**) Mass density $\rho_{\mathrm{mass}}$ = $\sum_{i}^{N}m_{i}/V_{\mathrm{cell}}$ obtained after NPT equilibration at 700 K and 1 bar for doped amorphous cells A${}_{1}$–A${}_{7}$ and completely Ti${}^{4+}$ depleted T${}_{0}$. (**b**) Respective atom densities with $\rho_{\mathrm{atom}}$ = $\sum_{i}^{N}\mathrm{atoms}/V_{\mathrm{cell}}$.

Exchanging Ti${}^{4+}$ for Mg${}^{2+}$ and Li${}^{+}$ leads to a steady increase of the total number of atoms. Yet, the equilibrated volume of these systems does not necessarily correlate linearly with this change in atom count as the composition is altered. While the mass density with higher doping concentrations shows as strictly decreasing trend as $m_{\mathrm{Ti}}$ > $m_{\mathrm{Mg}}$ + 2$m_{\mathrm{Li}}$, cf. Figure 11a, the total number density of all atoms reflects the observed dip in A${}_{5}$ and exhibits a non-linear behavior, cf. Figure 11b. It is important to note that doping effects, not surprisingly in particular for such amorphous phases, may not be predicted in a back-of-the-envelope fashion but require more detailed investigations.

The higher Al${}^{3+}$ content in T${}_{0}$ leads to a drastic increase in mass density. A concomitant introduction of excess Li${}^{+}$ to maintain charge neutrality when replacing tetravalent Ti${}^{4+}$ by Mg${}^{2+}$ and Al${}^{3+}$ leads to an increase also in atom density for T${}_{0}$.

### 3.6. Dopant Impact on Ion Dynamics

Due to the finite width of grain boundaries in the nano-scale regime, intricate experimental analyses are required to resolve Li ion conductivity of LATP grain boundaries from measurements of the bulk material conductivity. By means of electrochemical impedance spectroscopy Mertens et al. have identified the grain boundary conductivity to be multiple orders of magnitude lower than in the LATP grains [57]. The low Li ion conductivity is not necessarily inherent to the structural nature of the grain boundary phase itself but may be masked by poor interfacial contacting e.g., due to mechanical cracks or the formation of ionically insulating secondary phases [72,73]. MD simulations of the recently established atomistic grain boundary model suggest the orders of magnitude difference to be largely attributable to such microstructural defects [9]. Nonetheless, even in the idealized models investigated in previous works the amorphous bulk phase exhibits a lower Li ion conductivity [9] which may be increased by interfacial engineering.

**Figure 12:**
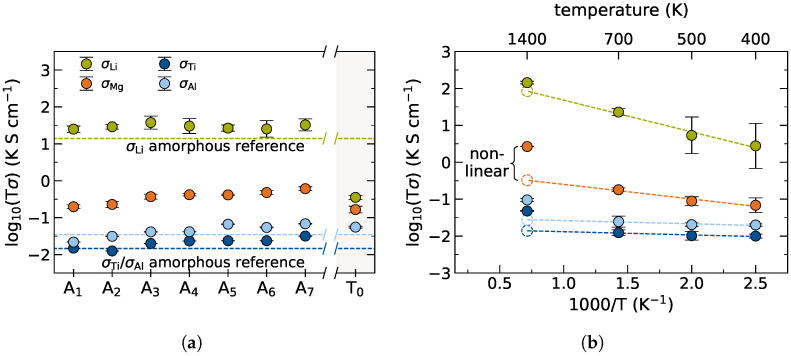
Ion conductivities of Mg${}^{2+}$ doped amorphous bulk cells. (**a**) Li${}^{+}$, Mg${}^{2+}$, Al${}^{3+}$, and Ti${}^{4+}$ conductivities as determined from 2 ns MD simulations at 700 K for amorphous bulk model ensembles A${}_{1}$–A${}_{7}$. The dashed lines indicate respective reference conductivities obtained from A${}_{\mathrm{ref}}$. Respective cationic conductivities of a doping realization T${}_{0}$, i.e., completely depleted of Ti${}^{4+}$, are shown in the right panel. (**b**) Arrhenius-type plot for cation conductivities in A${}_{4}$ as determined from 2 ns MD simulations at 400 K, 500 K, 700 K, and 1400 K, respectively.

Resulting cationic Li${}^{+}$, Mg${}^{2+}$, Al${}^{3+}$, and Ti${}^{4+}$ conductivities for the amorphous cells A${}_{1}$–A${}_{7}$, are obtained from MD simulations at 700 K for 2 ns and shown in Figure 12a. The Li conductivity is increased by 22–38% upon doping with divalent Mg${}^{2+}$ as compared to the reference undoped system. An enhancement of Li mobility in the grain boundary can be attributed to a locally higher concentration of charge carriers in the amorphous bulk domain, as already suggested by Mertens et al. [57]. The increase in Li${}^{+}$ ion conductivity however does not seem to correlate linearly with the doping concentration.

The investigated stoichiometry T${}_{0}$ which is completely depleted of Ti${}^{4+}$ yields a drastically decreased Li${}^{+}$ conductivity by 2 orders of magnitude. This no longer facilitates sufficient charge carrier diffusion for a bulk Li conducting electrolyte. However, the overall effect on performance also depends on the thickness of such an amorphous interphase. A possible reason for this drastic conductivity drop may be the formation of secondary phases which virtually do not contribute to Li ion conductivity. The presence of an AlPO${}_{4}$ secondary phase, for example, has been shown to significantly affect the LATP performance [72]. Similarly, Welsch et al. [74] have recently found a significantly lowered Li${}^{+}$ mobility in Li-Mg-phosphate glass networks. While this particular T${}_{0}$ stoichiometry studied herein is thus not a viable candidate for interfacial engineering, other possible compositions in the multidimensional phase space may exhibit less drastic effects. Since it is generally desirable to reduce the amount of interfacial TM centers while maintaining a level of Li${}^{+}$ conductivity that is still sufficient for a thin intergranular film, further stoichiometries should be systematically screened in the future.

The dopant mobility in the amorphous domain for A${}_{1}$–A${}_{7}$ is about 2 orders of magnitude lower than Li${}^{+}$, cf. Figure 12a. As established previously, for true interfacial engineering the Mg${}^{2+}$ dopant needs to stay locally confined in the amorphous bulk regime, thus requiring a low dopant mobility. While the dopant conductivity is considerably lower than the charge carrier conductivity, the trend suggests an increasing Mg${}^{2+}$ mobility with increasing doping concentration. For other doping realizations the Mg${}^{2+}$ mobility therefore needs to be closely monitored, as it is not straight-forward to extrapolate such trends.

Both, Al${}^{3+}$ and Ti${}^{4+}$ mobility increase only marginally from the reference conductivity and remain about 3 orders of magnitude lower than the Li${}^{+}$ conductivity. The immobility of the screened host system manifests the solid character of the chosen Mg${}^{2+}$ doped interphase stoichiometry even at elevated temperatures of 700 K. This is a general requirement for any SSE material under operating conditions. During high temperature synthesis, e.g., sintering, however, the interphase needs to exhibit higher mobility for sufficient densification and satisfying contacting between phases. Cation conductivities of A${}_{4}$ retrieved from 2 ns MD simulations at 400 K, 500 K, 700 K, and 1400 K, shown in Figure 12b, validate a higher ion mobility in the sintering temperature regime >1000 ${}^{\circ }$C beyond linear extrapolation. This suggests successful electrolyte processibility for the introduced doped interphase stoichiometries.

## 4. Discussion

In our previous work we have established a novel approach towards atomistic modelling of a realistic extended grain boundary in LATP, by leveraging the direct combination of experimental TEM and APT data with computational simulations. Besides a highly amorphous grain boundary interphase, the LATP grains are encapsulated by a protective nanoscale complexion. With a self-limiting width in the low nanometer regime and distinctively different structural and chemical features from adjacent phases, this newly identified motif is considered a 2D nanostructure. In a consequential next step we herein actively engineer the electrolyte interphase via aliovalent doping to further exploit and enhance the advantageous grain boundary properties. Encouraged by local accumulation of a Mg${}^{2+}$ impurity observed in our earlier APT analysis, we focus on this divalent cation as interfacial dopant for LATP.

To do so, seven different doping concentrations are realized by charge neutral substitution of higher valent cations to enrich the amorphous bulk phase with Mg${}^{2+}$ and, in particular, deplete it of the problematic Ti${}^{4+}$. Employing an MC swapping protocol based on replica exchange to enhance statistical sampling, we find that the Mg${}^{2+}$ does not bleed heavily into the adjacent crystalline grain domains thus identifying Mg${}^{2+}$ as a suitable dopant for true interfacial engineering.

A more in depth analysis of the minimal leakage of Mg${}^{2+}$ ions into the grain suggests preferential substitution in the Ti/Al host framework as compared to the Li sites. Only at higher doping concentrations we do find an infiltration of Mg${}^{2+}$ ions into the Li ion channels. Extensive MD simulations confirm that furthermore no severe compromising of Li ion conductivity is expected as the interconnected 3D Li network in LATP is not impaired by localized, single ion Mg${}^{2+}$ clogging.

Analyses conducted on amorphous bulk cells at different Mg${}^{2+}$ doping concentrations substantiate the qualification of divalent Mg${}^{2+}$ as interfacial dopant since an improvement of interphase properties is observed. Especially a lowered Ti${}^{4+}$ content may lead to considerably lower residual electronic conductivity via polaron hopping and thus protect the electrolyte from degradation. Furthermore, a concomitant local increase in Li${}^{+}$ charge carrier concentration with higher Mg${}^{2+}$ doping in the interphase leads to improved Li${}^{+}$ ion conductivity.

In particular for LATP, the formation of extended grain boundaries beyond the simulated length scale is known [57], highlighting the importance of bulk amorphous interphase properties. Active interfacial engineering may open degrees of freedom for the microstructural design, e.g., by filling cracks leading to better contacting and thus possibly improving electron impedance and furthermore exploit advantageous features of nanostructured motifs.

As has been shown by comparison of different relevant stoichiometries that were analyzed in detail, the effect of doping on electrolyte performance is in no way straightforward and linear trends cannot be simply assumed, in particular not for the investigated amorphous interphases. Non-trivial effects such as density changes and the generally known formation of secondary phases [72] need to be taken into account. Going forward, experimental input is therefore essential to reduce the vast design space of possible interphase stoichiometries for this quinary system and to yield meaningful validated candidates.

In conclusion, we herein propose Mg${}^{2+}$ to be a promising candidate for interfacial doping in LATP as it does not interfere with the overall electrolyte performance while locally improving the critical grain boundary properties. Conceptually, our work presents a novel computational approach to assess the suitability of a specific electrolyte interfacial dopant for a realistic grain boundary model. Besides its high Li conductivity, LATP is especially promising as an SSE candidate due to the natural abundance of its elements and its stability against contact with water and air. The latter may facilitate the experimental realization of coating LATP grains with computationally predicted interfacial stoichiometries, e.g., through atomic layer deposition as established for coating in electrolyte/electrode interfaces [75,76,77]. An alternative synthesis method may be the wet impregnation of mother powder which is well known in heterogeneous catalysis and has recently been introduced also in the fabrication of electrodes in solid oxide fuel cells [78]. As a general experimental synthesis recommendation, Mg${}^{2+}$ dopant concentrations are suggested between 0.8 at% as the lower limit observed in APT measurements, and 6.1 at% as an upper limit realized in the completely Ti depleted stoichiometry. Such dry or incipient wetness impregnation may be a promising approach [79] to coating the LATP grains prior to sintering. The experimental sintering temperature of 1100 ${}^{\circ }$C as originally applied in the LATP synthesis [71] should not be exceeded. Such rather intricate synthesis protocols demand for a narrowing of the vast design space of possible interphase stoichiometries to only a few confidently predicted candidates. Due to non-trivial effects upon doping however, simple large-scale screening, e.g., grid search, is not applicable. Instead, future leveraging of experimental insights and computational prediction in an adaptive Design of Experiment or Bayesian optimization ansatz will be needed.

## Data Availability

The data presented in this study are available on request from the corresponding author.

## Supplementary Materials

The following supporting information can be downloaded at https://www.mdpi.com/article/10.3390/nano12172912/s1, Figure S1: Local optimization of Mg${}^{2+}$-Phosphate interaction parameters in Mg${}_{3}$(PO−4)${}_{2}$, Figure S2: Energy (top) and force (bottom) correlations computed with an extended classical force field and reference DFT data resolved for three stoichiometries according to Li${}_{1+x+2y}$Al${}_{x}$Mg${}_{y}$Ti${}_{2-x-y}$(PO${}_{4}$)${}_{3}$, Figure S3: Ion dynamics in crystalline Li${}_{1.6}$Al${}_{0.4}$Mg${}_{0.1}$Ti${}_{1.5}$(PO${}_{4}$)${}_{3}$ (LAMTP), Figure S4: Schematic Monte-Carlo (MC) swapping protocol. Table S1: Mg${}^{2+}$ and Buckingham $\rho_{\mathrm{Mg}-\mathrm{ion}}$ parameters from local optimization of Mg${}_{3}$(PO${}_{4}$)${}_{2}$ volume. References [80,81,82,83] are cited in the supplementary materials.

## Abbreviations

The following abbreviations are used in this manuscript:

LIB	Lithium Ion Battery
ASSB	All Solid-State Battery
SSE	Solid-State Electrolyte
LATP	Li${}_{1+x}$Al${}_{x}$Ti${}_{2-x}$(PO${}_{4}$)${}_{3}$
TEM	Transmission Electron Microscopy
APT	Atom Probe Tomography
TM	Transition Metal
MD	Molecular Dynamics
DFT	Density Functional Theory
MC	Monte-Carlo
MSD	Mean Square Displacement
NASICON	NA Super Ionic CONductor
NN	Nearest Neighbor

## Appendix A

**Table A1:** Parameter set obtained for the optimized core-shell Li${}_{1.3}$Al${}_{0.3}$Ti${}_{1.7}$(PO${}_{4}$)${}_{3}$ force field as introduced by Stegmaier et al. [9]. The herein extended Mg${}^{2+}$ parameters and Mg${}^{2+}$-ion Buckingham parameters are depicted in bold.

**Atom Types**											
**Species**				**Mass (u)**				**Charge (e)**			
	Ti ^1^			47.867				+2.196			
	Al			26.982				+1.647			
	Li ^2^			6.940				+0.549			
	P			30.974				+2.745			
	O${}_{\mathrm{core}}$ ^1^			15.698				+0.500			
	O${}_{\mathrm{shell}}$ ^1^			0.301				-1.598			
	**Mg**			**24.305**				**+1.098**			
**Buckingham Parameters**											
**species ij**			**A${}_{\mathrm{ij}}$ (eV)**			**$\rho_{\mathrm{ij}}$ (Å)**			**C${}_{\mathrm{ij}}$ (eVÅ${}^{6}$)**		
	Li-Li ^2^		38,533.955			0.100			0.000		
	Ti-Li ^2^		33,089.570			0.127			0.000		
	Ti-Ti ^1^		31,120.528			0.154			5.250		
	Li-O ^2^		15,465.549			0.167			0.000		
	O-O ^1^		11,782.885			0.234			30.220		
	Ti-O		18,448.156			0.194			12.590		
	P-P		53,210.800			0.284			0.000		
	P-O		32,397.875			0.155			7.831		
	P-Li		30,393.156			0.131			0.000		
	P-Ti		10,469.346			0.139			136.835		
	Al-Al		42,700.844			0.197			0.000		
	Al-Li		32,315.936			0.127			0.000		
	Al-Ti		10,489.082			0.131			6.862		
	Al-O		17,491.787			0.179			7.920		
	Al-P		10,580.062			0.137			114.906		
	**Mg-Mg**		**20,671.120**			**0.148**			**0.000**		
	**Mg-Li**		**24,050.700**			**0.138**			**0.000**		
	**Mg-Ti**		**12,947.270**			**0.117**			**6.200**		
	**Mg-O**		**55,835.770**			**0.179**			**12.620**		
	**Mg-P**		**56,162.410**			**0.130**			**120.000**		
	**Mg-Al**		**21,485.920**			**0.230**			**0.580**		
**Core-Shell Force Constants** ^3^											
**species ij**						**k${}_{\mathrm{ij}}$ (eVÅ${}^{-2}$)**					
O${}_{\mathrm{core}}$-O${}_{\mathrm{shell}}$						88.6					

^1^ Parameters adopted from TiO force field [34]. ^2^ Parameters adopted from LTO force field [84]. ^3^ Interaction potential form: *V_ij_* = 1/2*k_ij_r_ij_*.
