# Supplementary material for: Exploiting Nanoscale Complexion in LATP Solid-State Electrolyte via Interfacial Mg2+ Doping
Source: Nanomaterials (Basel). 2022 Aug 24;12(17):2912. doi: 10.3390/nano12172912 (PMC9457643; doi:10.3390/nano12172912)
Supplement: Supplementary file 1 [file nanomaterials-12-02912-s001.zip › nanomaterials-1832439-supplementary update.pdf]

nanomaterials

Supplementary File

Exploiting Nanoscale Complexion in LATP Solid-State Electrolyte via Interfacial Mg^{2+} Doping

Sina Stegmaier [1], Karsten Reuter [2] and Christoph Scheurer [2,3,*]

[1] Department of Chemistry, Technical University of Munich, 85747 Garching, Germany
[2] Theory Department, Fritz-Haber-Institut der Max-Planck-Gesellschaft, 14195 Berlin, Germany
[3] Institute of Energy and Climate Research, Fundamental Electrochemistry (IEK-9), Forschungszentrum Jülich GmbH, 52425 Jülich, Germany
* Correspondence: scheurer@fhi.mpg.de

S1. Force field extension by Mg^{2+}

S1.1. Mg^{2+}–phosphate parameterization in $Mg_3(PO_4)_2$

First, the Mg–phosphate interactions are locally optimized in the $Mg_3(PO_4)_2$[1] material. An atomistic structure featuring 1404 atoms is shown in Figure S1 (a). Initial values are chosen as arithmetic mean of Li^+ and Al^{3+} parameters, with

$$B_{\mathrm{Mg}^{2+},j} = \frac{B_{\mathrm{Li}^{+},j} + B_{\mathrm{Al}^{3+},j}}{2}. \tag{S.1}$$

Structural relaxation with these parameters leads to an underestimation of the cell volume as compared to the reference structure. Therefore, the repulsive contribution $\rho_{\text{Mg-ion}}$ in the Coulomb-Buckingham

$$U_{ij} = \frac{1}{4\pi\epsilon_0}\frac{q_i q_j}{r_{ij}} + A_{ij}\exp\left(-\frac{r_{ij}}{\rho_{ij}}\right) - \frac{C_{ij}}{r_{ij}^6}. \tag{S.2}$$

is optimized. A Limited-memory Broyden–Fletcher–Goldfarb–Shanno algorithm (L-BFGS) optimizer as implemented in the *SciPy* python library [1] is used to minimize the volume difference from the reference structure. Between optimization steps the structure is equilibrated in NPT at 300 K and 1 bar for 20 ps.

Partial radial distribution functions (RDFs) for the different ion pairs are shown in Figure S1 (b), which are obtained from NPT Molecular Dynamics (MD) simulations at 300 K and 1 bar for 50 ps. Thermal vibration during MD simulation leads to broadening of peaks as compared to the reference structure. The Mg-O and Mg-Mg profiles show higher agreement for the optimized parameters as compared to the initial guess. The larger average nearest neighbor distance of Mg-O leads to a better volume match with the reference structure.

[1] mp-14396 [2]

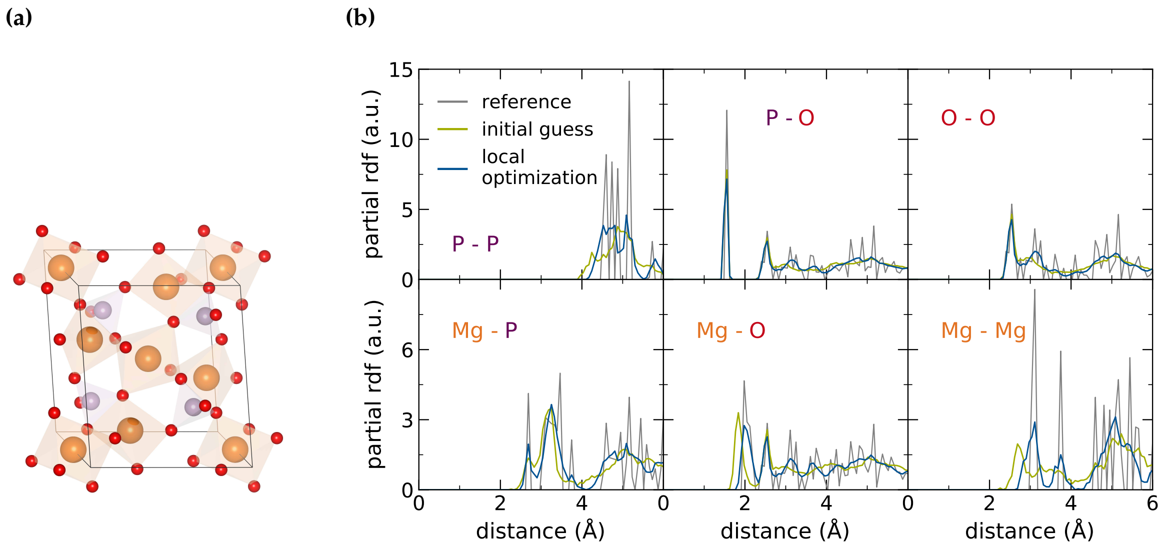

Figure S1. Local optimization of Mg^{2+}-Phosphate interaction parameters in $Mg_3(PO_4)_2$. **(a)** Atomistic structure of $Mg_3(PO_4)_2$ (ID: mp-14396 [2]). Elemental colors chosen as Mg ●, O ● and P ●. **(b)** Partial RDFs for for database reference structure, initial interaction parameters from arithmetic mean of Li^+ and Al^{3+} and locally optimized interaction parameters.

Table S1 provides the optimized $\rho_{\text{Mg-ion}}$ values. These serve as input values for subsequent global fitting of Mg^{2+}–ion interaction parameters.

Table S1. Mg^{2+} and Buckingham $\rho_{\text{Mg-ion}}$ parameters from local optimization of $Mg_3(PO_4)_2$ volume.

**species**	**mass (u)**	**charge (e)**	**radius (pm)**
Mg	24.305	+1.098	72.0
	**Buckingham Parameters**		
**species** ij	**A**$_{ij}$ **(eV)**	ρ_{ij} **(Å)**	**C**$_{ij}$ **(eVÅ**^{6}**)**
Mg-Mg	40617.399	0.152	0.000
Mg-O	16478.668	0.187	3.960
Mg-P	20486.609	0.133	57.453

S1.2. Mg^{2+} doped LATP parameters

A reference Mg doped LATP database is set up with both, crystalline and amorphous slabs of ∼220 atoms. Crystalline slabs are cut from the ICSD reference structure (ID: 253240) [3] and amorphous slabs are cut from the amorphous bulk of previously established grain boundary models [4]. Three stoichiometries are set up for different values of x and y in $Li_{1+x+2y}Al_xMg_yTi_{2-x-y}(PO_4)_3$ (LAMTP), each with 25 crystalline and 25 amorphous stochastically doped structures. As a results, a reference database with a total of 150 structures is built.

Potential energy and atomic force vectors are obtained from Single Point Density Functional Theory (DFT) calculations utilizing the pseudo-potential plane wave code CASTEP [5], the GGA-level PBE functional [6], and ultrasoft pseudopotentials as provided by the GBRV library [7]. Previously converged settings are adopted with a plane wave cutoff energy of 750 eV and a Monkhorst-Pack grid density [8] of 0.07 Å^{-1}.

A Particle Swarm Optimizer (PSO), as implemented in the *inspyred* python module [9], is used for global optimization. A population size of 200 and a number of generations of 500 is chosen. The scoring function for the potential energy is given as:

$$\Delta E = \frac{\sqrt{\sum_{s,s'>s}\left[\left(E_s^{DFT} - E_{s'}^{DFT}\right) - \left(E_s^{FF} - E_{s'}^{FF}\right)\right]^2}}{\sqrt{\sum_{s,s'>s}\left[E_s^{DFT} - E_{s'}^{DFT}\right]^2}}, \tag{S.3}$$

where s runs over the structures in the training set. The scoring function for the atomic forces is given as:

$$\Delta F = \frac{\sqrt{\sum_{s,\alpha,\beta}\left[F^{DFT}_{s,\alpha,\beta} - F^{FF}_{s,\alpha,\beta}\right]^2}}{\sqrt{\sum_{s,\alpha,\beta}\left[F^{DFT}_{s,\alpha,\beta}\right]^2}}, \tag{S.4}$$

where α runs over the atoms in a specific structure and β over the cartesian coordinates x, y and z. Elemental weights other than Mg are set to zero and forces are weighted higher than energies for matching with $w_F = 10\, w_E$. A subsequent local optimization utilizing the previously described L-BFGS optimizer is performed. The final Buckingham parameters are listed in Table A1 in the manuscript.

S1.3. Validation of extended force field

Correlation plots of force field potential energies and forces are shown in Figure S2 for three stoichiometries. All stoichiometries exhibit two clusters of energetically lower, crystalline Mg^{2+} doped LATP and energetically higher, amorphous Mg^{2+} doped LATP structures. Correlation of the potential energies for these high order system is in general agreement with DFT reference. For the amorphous slabs a slight overestimation of energies is observed for higher energy values. Considering the low Mg^{2+} content as dopant and the constraints from the majority of atom species other than Mg, the force correlation is sufficient for this classical force-field type.

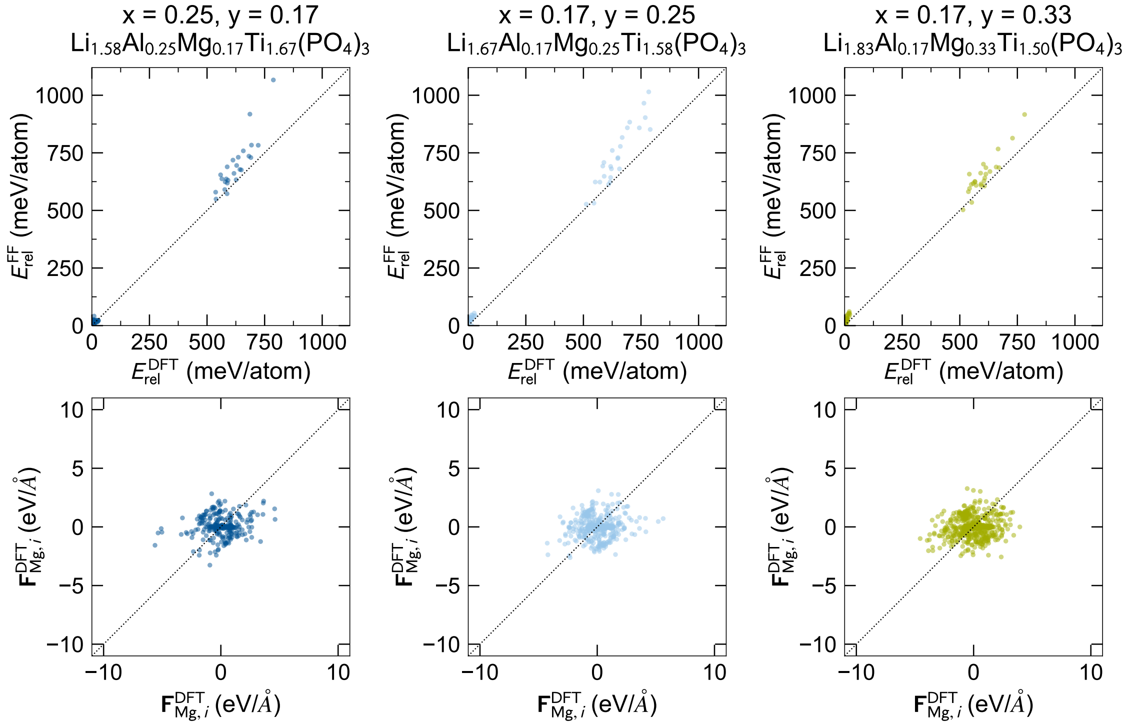

Figure S2. Energy (top) and force (bottom) correlations computed with an extended classical force field and reference DFT data resolved for three stoichiometries according to $Li_{1+x+2y}Al_xMg_yTi_{2-x-y}(PO_4)_3$.

Validation of Li ion conductivity against experimental data proves difficult, since Mg^{2+} doping for the LATP has not been reported to the best of our knowledge. Therefore, ion dynamics are validated against *ab initio* of the structurally very similar (NA-Super-Ion-CONductor) NASICON-type $Li_{1+x}Al_xGe_{2-x}(PO_4)_3$ (LAMGP), as recently studied by Nikodimos et al. [10]. A crystalline LAMTP cell with $x = 0.4$ and $y = 0.1$ is constructed with 75 % Ti sites occupied by Ti^{4+}, 20 % sites occupied by Al^{3+} and 5 % occupied by Mg^{2+} yielding 893 atoms. Five such structures are constructed to account for stochastic sampling of ions. MD simulations are run for each structure at 300 K, 400 K, 500 K, 700 K and 1400 K. Equilibration is performed at 1 bar and the respective temperature for 50 ps and production runs are conducted in NVT at the respective temperature for 500 ps.

LAMTP elemental Mean-Square Displacements (MSDs) are shown in Figure S3 (a) at 700 K. The mobility trend qualitatively reproduces the trend reported by Nikodimos et al. [10] with

$$Li^{+} \gg O^{2-} > Mg^{2+} > P^{5+} \simeq Al^{3+} \simeq Ti^{4+}/Ge^{4+}.$$

An Arrhenius-type plot is shown in Figure S3 (b) for Li ion diffusion of LAMTP and LAMGP at different temperatures. While structurally similar, the undoped NASICON-type materials show different bulk Li ion conductivity with LATP $3.0 \cdot 10^{-3}$ $S \cdot cm^{-1}$ [11] and LAGP $3.38 \cdot 10^{-4}$ $S \cdot cm^{-1}$ [12]. LATP charge carrier mobility is thus faster by 1.42 orders of magnitude as compared to LAGP. MD simulations with the extended force field reproduce this difference with 1.44 orders of magnitude higher LAMTP conductivity as compared to LAMGP [10]. An extension by Mg^{2+} interaction parameters of the LATP classcial force field shows satisfactory agreement with the limited available *ab initio* reference data [10].

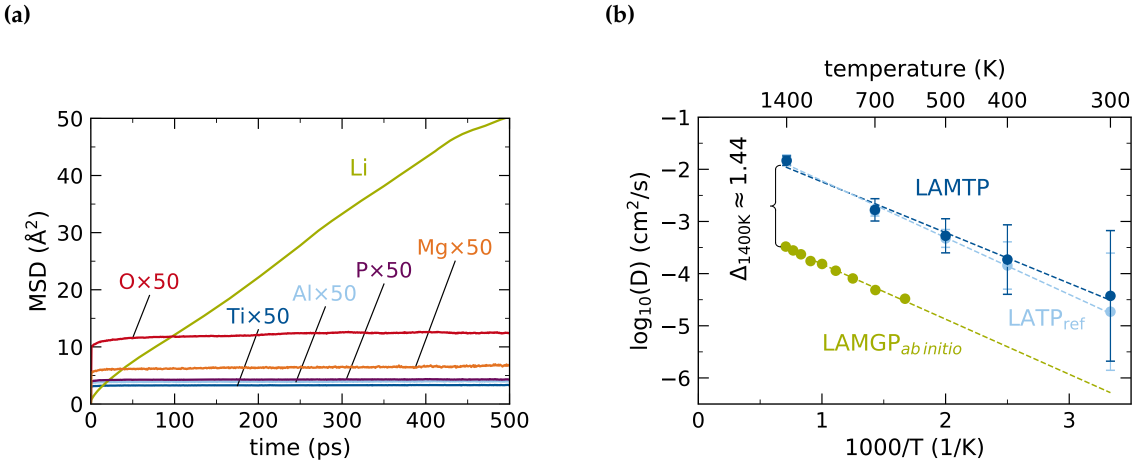

Figure S3. Ion dynamics in crystalline $Li_{1.6}Al_{0.4}Mg_{0.1}Ti_{1.5}(PO_4)_3$ (LAMTP). **(a)** Elemental MSDs for LAMTP at 700 K. MSDs for immobile species are magnified by a factor of 50. **(b)** Arrhenius-type plot with Li diffusion coefficients as determined from MD simulations. Also shown are *ab initio* values from Nikodimos et al. [10] for $Li_{1.6}Al_{0.4}Mg_{0.1}Ge_{1.5}(PO_4)_3$ (LAMGP) and respective undoped LATP reference values.

S2. Monte-Carlo swapping protocol

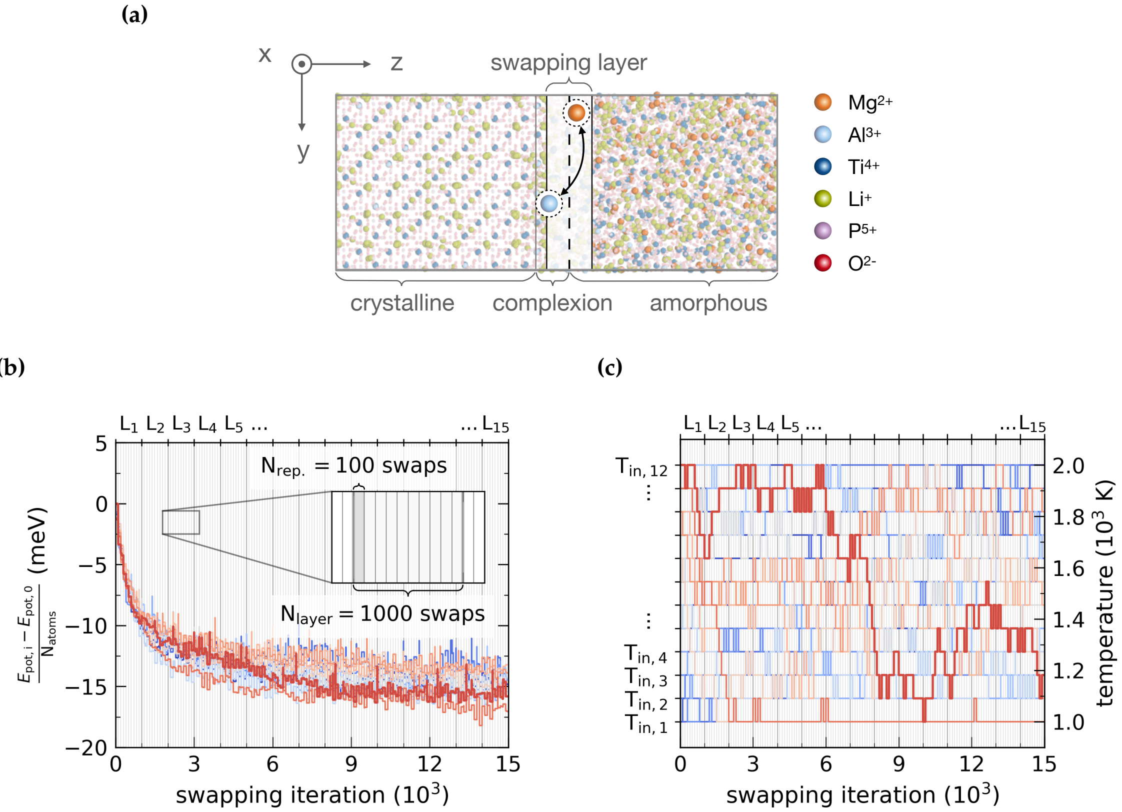

Figure S4. Schematic Monte-Carlo (MC) swapping protocol. **(a)** Attempted swap of $Mg^{2+} \leftrightarrow Ti^{4+}$ across the interface in first swapping layer. **(b)** Exemplary energy profiles of 12 randomly initialized walkers. Walker potential energies converge over the course of 15,000 attempted swaps. After $N_{rep} = 100$ swaps an NPT simulation is performed. After $N_{layer} = 1000$ swaps the swapping regime is extended by the next adjacent layer L_{i+1}. **(c)** Parallel tempering for the 12 exemplary walkers with different initial temperatures $T_{in,i}$ in an equidistant range between 1000 – 2000 K.

References

1. Zhu, C.; Byrd, R.H.; Lu, P.; Nocedal, J. Algorithm 778: L-BFGS-B: Fortran Subroutines for Large-Scale Bound-Constrained Optimization. *ACM Trans. Math. Softw.* **1997**, *23*, 550–560. https://doi.org/10.1145/279232.279236.
2. Jain, A.; Ong, S.P.; Hautier, G.; Chen, W.; Richards, W.D.; Dacek, S.; Cholia, S.; Gunter, D.; Skinner, D.; Ceder, G.; et al. Commentary: The materials project: A materials genome approach to accelerating materials innovation. *APL Mater.* **2013**, *1*, 011002. https://doi.org/10.1063/1.4812323.
3. Allen, F.H. The Development, Status and Scientific Impact of Crystallographic Databases. *Acta Crystallogr.* **1998**, *A54*, 758–771. https://doi.org/10.1107/S0108767398010563.
4. Stegmaier, S.; Schierholz, R.; Povstugar, I.; Barthel, J.; Rittmeyer, S.P.; Yu, S.; Wengert, S.; Rostami, S.; Kungl, H.; Reuter, K.; et al. Nano-Scale Complexions Facilitate Li Dendrite-Free Operation in LATP Solid-State Electrolyte. *Adv. Energy Mater.* **2021**, *11*, 2100707. https://doi.org/10.1002/aenm.202100707.
5. Clark, S.J.; Segall, M.D.; Pickard, C.J.; Hasnip, P.J.; Probert, M.I.; Refson, K.; Payne, M.C. First principles methods using CASTEP. *Z. Kristallogr.* **2005**, *220*, 567–570. https://doi.org/10.1524/zkri.220.5.567.65075.
6. Perdew, J.P.; Burke, K.; Ernzerhof, M. Generalized gradient approximation made simple. *Phys. Rev. Lett.* **1996**, *77*, 3865–3868. https://doi.org/10.1103/PhysRevLett.77.3865.
7. Garrity, K.F.; Bennett, J.W.; Rabe, K.M.; Vanderbilt, D. Pseudopotentials for high-throughput DFT calculations. *Comput. Mater. Sci.* **2014**, *81*, 446–452. https://doi.org/10.1016/j.commatsci.2013.08.053.
8. Monkhorst, H.J.; Pack, J.D. Special points for Brillouin-zone integrations. *Phys. Rev. B* **1976**, *13*, 5188–5192. https://doi.org/10.1103/PhysRevB.13.5188.
9. Tonda, A. Inspyred: Bio-inspired algorithms in Python. *Genet. Program. Evolvable Mach.* **2020**, *21*, 269–272. https://doi.org/10.1007/s10710-019-09367-z.

10. Nikodimos, Y.; Abrha, L.H.; Weldeyohannes, H.H.; Shitaw, K.N.; Temesgen, N.T.; Olbasa, B.W.; Huang, C.J.; Jiang, S.K.; Wang, C.H.; Sheu, H.S.; et al. A new high-Li^+-conductivity Mg-doped $Li_{1.5}Al_{0.5}Ge_{1.5}(PO_4)_3$ solid electrolyte with enhanced electrochemical performance for solid-state lithium metal batteries. *J. Mater. Chem. A* **2020**, *8*, 26055–26065. https://doi.org/10.1039/d0ta07807g.
11. Aono, H.; Sugimoto, E.; Sadaoka, Y.; Imanaka, N.; Adachi, G.y. Ionic Conductivity of Solid Electrolytes Based on Lithium Titanium Phosphate. *J. Electrochem. Soc.* **1990**, *137*, 1023–1027. https://doi.org/10.1149/1.2086597.
12. Zhang, M.; Huang, Z.; Cheng, J.; Yamamoto, O.; Imanishi, N.; Chi, B.; Pu, J.; Li, J. Solid state lithium ionic conducting thin film $Li_{1.4}Al_{0.4}Ge_{1.6}(PO_4)_3$ prepared by tape casting. *J. Alloys Compd.* **2014**, *590*, 147–152. https://doi.org/10.1016/j.jallcom.2013.12.100.
